# A candidate gene identified in converting platycoside E to platycodin D from *Platycodon grandiflorus* by transcriptome and main metabolites analysis

**DOI:** 10.1038/s41598-021-89294-1

**Published:** 2021-05-07

**Authors:** Xinglong Su, Yingying Liu, Lu Han, Zhaojian Wang, Mengyang Cao, Liping Wu, Weimin Jiang, Fei Meng, Xiaohu Guo, Nianjun Yu, Shuangying Gui, Shihai Xing, Daiyin Peng

**Affiliations:** 1 School of Pharmacy, Anhui University of Chinese Medicine, Hefei, 230012 China; 2 Institute of Traditional Chinese Medicine Resources Protection and Development, Anhui Academy of Chinese Medicine, Hefei, 230012 China; 3 College of Humanities and International Education Exchange, Anhui University of Chinese Medicine, Hefei, 230012 China; 4 College of Life Sciences and Environment, Hengyang Normal University, Hengyang, 421008 Hunan China; 5 Anhui Province Key Laboratory of Pharmaceutical Preparation Technology and Application, Anhui University of Chinese Medicine, Hefei, 230012 China; 6 Anhui Province Key Laboratory of Research and Development of Chinese Medicine, Hefei, 230012 China; 7 Synergetic Innovation Center of Anhui Authentic Chinese Medicine Quality Improvement, Hefei, 230038 China

**Keywords:** Molecular biology, Plant sciences

## Abstract

Platycodin D and platycoside E are two triterpenoid saponins in *Platycodon grandiflorus*, differing only by two glycosyl groups structurally. Studies have shown β-Glucosidase from bacteria can convert platycoside E to platycodin D, indicating the potential existence of similar enzymes in *P*. *grandiflorus*. An L_9_(3^4^) orthogonal experiment was performed to establish a protocol for calli induction as follows: the optimal explant is stems with nodes and the optimum medium formula is MS + NAA 1.0 mg/L + 6-BA 0.5 mg/L to obtain callus for experimental use. The platycodin D, platycoside E and total polysaccharides content between callus and plant organs varied wildly. Platycodin D and total polysaccharide content of calli was found higher than that of leaves. While, platycoside E and total polysaccharide content of calli was found lower than that of leaves. Associating platycodin D and platycoside E content with the expression level of genes involved in triterpenoid saponin biosynthesis between calli and leaves, three contigs were screened as putative sequences of β-Glucosidase gene converting platycoside E to platycodin D. Besides, we inferred that some transcription factors can regulate the expression of key enzymes involved in triterpernoid saponins and polysaccharides biosynthesis pathway of *P. grandiflorus*. Totally, a candidate gene encoding enzyme involved in converting platycoside E to platycodin D, and putative genes involved in polysaccharide synthesis in *P. grandiflorus* had been identified. This study will help uncover the molecular mechanism of triterpenoid saponins biosynthesis in *P*. *grandiflorus*.

## Introduction

*Platycodon grandiflorus* (Jacq.) A. DC., a perennial herb, is the sole species in genus Platycodon within the Campanulaceae family. The flowers of *P*. *grandiflorus* are blue purple or white, which can be used for ornamental and horticultural purpose. The root (platycodi radix) of *P*. *grandiflorus* has diverse pharmacological activities and can be used for the treatment of some chronic inflammatory diseases such as asthma, bronchitis and tuberculosis^1^. The dried form of the platycodi radix is officially listed as a traditional herbal medicine in the Chinese, Korean and Japanese Pharmacopoeia^2^. It is also being pickled in northeast China, and made into kimchi in the Korean Peninsula. It has medicinal, edible, ornamental value in one, with immeasurable development prospects.

As a traditional Chinese herb, *P*. *grandiflorus* is a rich source of natural secondary metabolic products that have various chemical structural types. More than 100 compounds have been isolated from *P*. *grandiflorus*, including steroidal saponins, flavonoids, phenolic acids, polyacetylenes, and sterols, etc.^3^. Triterpenoid saponins are the main active components in *P*. *grandiflorus*, including platycodin D (PD), platycoside E (PE), platycodigenin and platyconic acid A, etc*.* PD is one of the major triterpenoid saponins with higher pharmacological activity than the other platycosides from platycodi radix, and have multiple pharmacological effects, such as immunostimulation^4^, anti-inflammation^5^, anti-obesity^6^, anti-atherosclerosis^1^, and anticancer^7^. The structure of platycoside E is similar to that of platycodin D, and both of them are oleanane-type triterpenoid saponin. PE has two additional glucose groups compared to PD^8^. PE could convert to PD through the hydrolysis action of a de-glucosidase. *P*. *grandiflorus* polysaccharides (PGPs) are another important active component in this medicinal plant, and studies have confirmed that PGPs are involved in antioxidant activity^9^, it can activate macrophage and enhance non-specific immunity function^10^. A research has shown that a selenium polysaccharide from the platycodi radix may be considered as a potential and useful antioxidant agent^9^.

Pentacyclic triterpenoid saponins have been well-known as important secondary metabolites in plants. 2, 3-oxidosqualene, a direct precursor of triterpenoid saponins, is synthesized mainly by the mevalonic acid (MVA) pathway^11^. However, the operator of the MVA pathway in regulating the biosynthesis of triterpenoids even phytosterols in *P*. *grandiflorus* has not been clearly described^12^. Farnesyl pyrophosphate (farnesyl-PP) is synthesized from isopentenyl diphosphate (IPP) and dimethylallyl diphosphate (DMAPP) under the catalysis of farnesyl pyrophosphate synthase (FPP)^11,13^. Under the catalysis of enzymes such as squalene synthase (SS) and squalene epoxidase (SE), 2,3-oxidosqualene is produced thereafter^14^. Subsequently, triterpenoids saponins synthesized from 2,3-oxidosqualene by three kinds of enzymes which are oxidized squalene cyclase, cytochrome P450 monooxygenase and uridine diphosphate-dependent glycosyltransferase. In other words, 2,3-oxidosqualene is converted to polygalacic acid, platycodigenin and platycogenic acid A by successive enzymes such as β-amyrin synthase (β-AS), β-amyrin 28-oxidase (β-A28O) and a series of cytochrome P450 (CYPs)^15^. Subsequently, the conversion of polygalacic acid into polygalacin D, platycodigenin into platycoside E, and platycogenic acid A into platyconic acid A are all catalyzed by certain kinds of GTs (Glycosyltransferases)^16^. Platycodin D, an oleanane-type triterpenoid saponin, is the main bioactive component and has stronger pharmacological activities, but little is clear on its biosynthesis in *P*. *grandiflorus* at present. It has been found that platycoside E is a precursor of platycodin D, and PE can be converted to PD by enzyme catalysis. Their biological activities can be increased and their bioavailability and cell permeability would be improved due to their reduced size resulted by de-glycosylation of saponins^17^.

**Figure 1:**
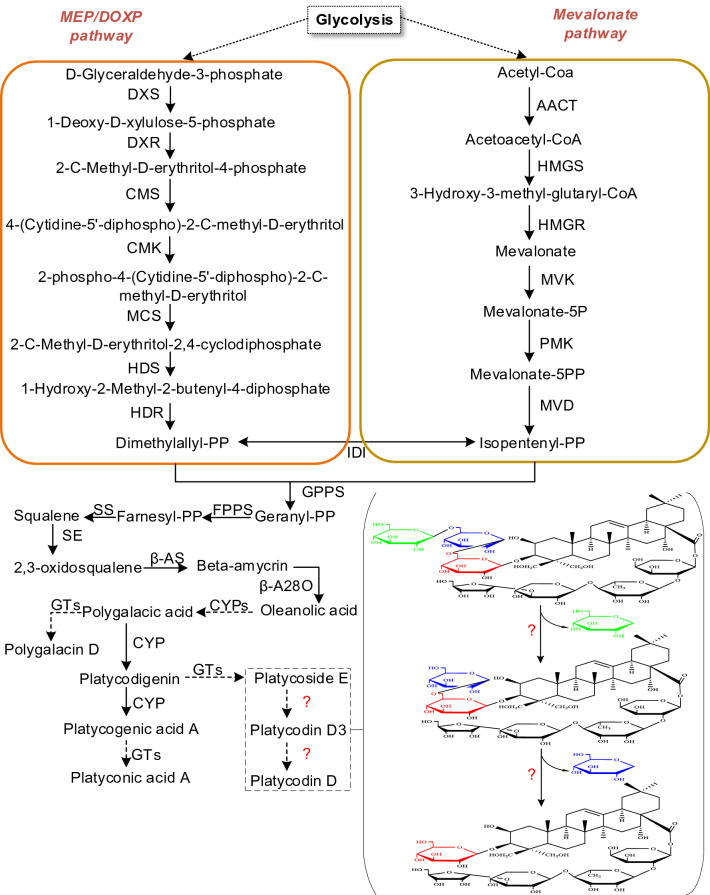
Triterpenoid saponin biosynthetic pathways predicted in *P*. *grandiflorus*. Arrows with solid lines represent the identified enzymatic reactions, and arrows with dashed lines represent multiple enzymatic reactions through multiple steps and putative enzymatic reactions.

From the chemical structure of platycodin D and platycoside E, it can be predicted that there are enzymes which can catalyze degradation of glycosyl group from PE to PD. Two extracellular experiments showed that β-D-glucosidase from *Aspergillus usamii* and *Caldicellulosiruptor bescii* can successfully catalyze conversion of PE and platycodin D3 into PD under optimal reactions conditions^18,19^. In this study we attempt to find out appropriate candidate genes encoding enzymes involving in conversion of PE, platycodin D3 into PD in traditional Chinese medicinal plant *P. grandiflorus*. The pathway of triterpenoid saponins in *P*. *grandiflorus* is predicted referring to the terpenoid backbone and saponin biosynthesis in KEGG^20^ (https://www.kegg.jp/dbget-bin/www_bget?map00900), as shown in Fig. 1.

Polysaccharides are extremely important bio-macromolecules, and have a wide range of industrial value and clinical role^21^. There are enormous types of polysaccharides in *P*. *grandiflorus*, named as *P*. *grandiflorus* polysaccharides (PGPs), which have important pharmacological activities. However, few relevant studies and analyses on its biosynthetic pathways were reported. Sucrose is firstly produced in plants through photosynthesis in plant chloroplast. Sucrose generates UDP-Glc in the presence of sucrose synthase (SUS), and then UDP-Glc is converted into GDP-Glc under successive catalytic reactions, including UDP-sugar pyrophosphorylase (USP)^22^, UDP-glucose phosphorylase (UGP), GDP-glucose pyrophos-phorylase (GGP)^23^. Sucrose-6P is biosynthesized from sucrose under the catalysis of sucrose-specific enzyme II (ScrA)^24,25^. Subsequently, a series of enzymes such as β-Fructofuranosidase (SacA), Mannose-6-phosphate isomerase^11^, Mannose-1-phosphate Guanylyl-transferase (GMPP) and other enzymes catalyze sucurose-6P to generate GDP-Man. Then, GDP-Fuc is synthesized from GDP-Man by enzymes such as GDP-Mann 4,6-dehydratase (GMDS) and GDP-L-Fucose (TSTA3)^26,27^. In addition, through the catalysis of nucleotide-diphospho-sugar (NDP-sugar) interconversion enzymes (NSEs), many other NDP sugars were produced from UDP-Glc and GDP-Man^28^. Another way is that sucrose invertase (INV) and hexokinase (HK) catalyze the production of D-Glucose-6P from sucrose^29^. Subsequently, D-Glucose-6P is converted into UDP-Gal, UDP-GalA, UDP-Rha and other polysaccharide precursors under the catalysis of enzymes such as Phosphoglucomutase (PGM), UTP-glucose-1-phosphate Uridylyltransferase (UGP2), and UDP-apiose/xylose synthase (AXS) et al^30^. Finally, through the catalysis of various glycosyltransferases (GTs), the active monosaccharide unit NDP-sugar is added to the sugar residues of various polysaccharides and glycoconjugates^26^. The biosynthesis pathway of polysaccharides in *P*. *grandiflorus* was inferred as shown in Supplementary Figure S1.

RNA-Seq has been widely used to analyze the pathway of secondary metabolite biosynthesis and regulation, excavate key enzyme genes and transcription factors in medicinal plants^31,32^, which provides a scientific basis for the efficient accumulation and effective utilization of active ingredients in medicinal plants. Transcriptome sequencing have been completed in many medicinal plants such as *Taxus chinensis*^33^, *Panax quinquefolius*^34^, *Coptis chinensis*^35^, *Panax zingiberensis*^15^, and many other medicinal plants. Many functional genes are discovered and partial specific biosynthetic pathways of secondary metabolite are analyzed.

In this paper, calli of *P*. *grandiflorus* were induced by tissue culture techniques. The contents of PE, PD and PGPs in calli and each organ of original plant of *P*. *grandiflorus* were determined by high performance liquid chromatography (HPLC) and Phenol–sulfuric acid method, respectively. Based on the differences of the secondary metabolites between calli and organs of original plant *P*. *grandiflorus*, RNA-seq was performed between calli and leaves. Comparative analysis of the transcriptome data provides valuable resources for further studies of the molecular mechanisms of terpenoids, saponins and polysaccharides biosynthesis in *P*. *grandiflorus*. In this study, the differences between calli and organs of original plants were analyzed, and candidate key genes and transcription factors were identified to help our knowledge of the metabolism and regulation of secondary metabolites in *P*. *grandiflorus*.

## Results

### Calli induction of *Platycodon grandiflorus*

The effects of four explants, different basic media, and plant growth regulator combinations on calli induction of *P*. *grandiflorus* were studied by orthogonal experimental design of 4 factors and 3 levels (L_9_(3^4^)) without consideration of interactions among factors. Each treatment has 60 independent duplicates (20 bottles with 3 pieces). The calli induced are shown in Supplementary Figure S2, and the results are shown in Table 1.

**Table 1:** Orthogonal experiment (L_9_(3^4^)) analyzing the effects of different factors on callus induction of *P*. *grandiflorus*.

	Factors				Inoculation Num (non-contaminated)	Calli Num	Induction rate (%)
	Media (A)	Explants (B)	NAA mg/L (C)	6-BA mg/L (D)			
1	B5	Leaves	0.1	0.5	56	4	7.14
2	B5	Stems with nods	0.2	1.0	58	22	37.93
3	B5	Stems	1.0	2.0	57	8	14.04
4	MS	Leaves	0.2	2.0	53	6	11.32
5	MS	Stems with nods	1.0	0.5	57	57	100.00
6	MS	stems	0.1	1.0	54	2	3.70
7	WPM	leaves	1.0	1.0	55	0	0.00
8	WPM	Stems with nods	0.1	2.0	58	23	39.66
9	WPM	stems	0.2	0.5	56	4	7.14
k1	59.11	18.46	50.50	114.28			
k2	115.02	177.59	56.39	41.63			
k3	46.80	24.88	114.04	65.02			
K1	19.70	6.15	16.83	38.09			
K2	38.34	59.20	18.80	13.88			
K3	15.60	8.29	38.01	21.67			
R	22.74	53.05	21.18	24.21			

The induction rates of the calli were calculated with the number of the calli divided by the number of uncontaminated explants. The K value and R value were obtained by statistical analysis of orthogonal experiment data, as the K value was the average of the rates from each level, and the R value called extreme difference is the difference between the maximum and minimum average values of each factor.

By analysis of variance (ANOVA), a significant difference among these factors and their levels (F = 8.67 > F_0.01_(3, 4) = 6.59) was identified. It can be concluded from the R value that the category of explants had a great influence on the calli induction, and stem with nodes is the optimal explants. It also can be concluded that the MS medium is better than the other two basic media and 6-BA could get calli induction more efficiently than NAA, and the optimal concentrations are 0.5 mg/L and 1.0 mg/L, respectively. Based on the above analysis, the best combination is A2B2C3D1. In order to verify whether the A2B2C3D1 is the best combination or not, a total of 30 stems with nodes were inoculated in 10 bottles with 3 duplicates per bottle, a total of 25 calli were induced after 50 days, and the induction rate is up to 83.33%.

It can be concluded from what is stated above that the best protocol for calli induction of *P*.*grandiflorus* is to use stem with nodes as the explants, and take MS + NAA 1.0 mg/L + 6-BA 0.5 mg/L as the optimal formula.

### Comparative of PD and PE content in organs and calli

**Figure 2:**
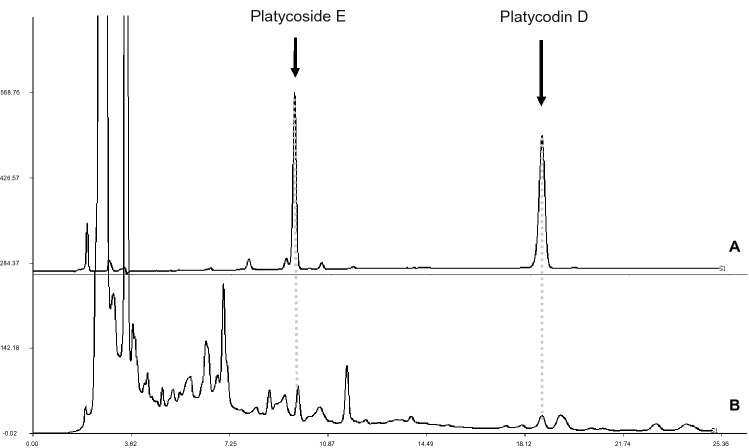
HPLC peak profiles of (**A**) standard products of PD and PE, and of (**B**) the *P*. *grandiflorus* extract. The X axis represents the retention time (min) of peaks, and the Y axis represents the height of the peak (mAU).

**Figure 3:**
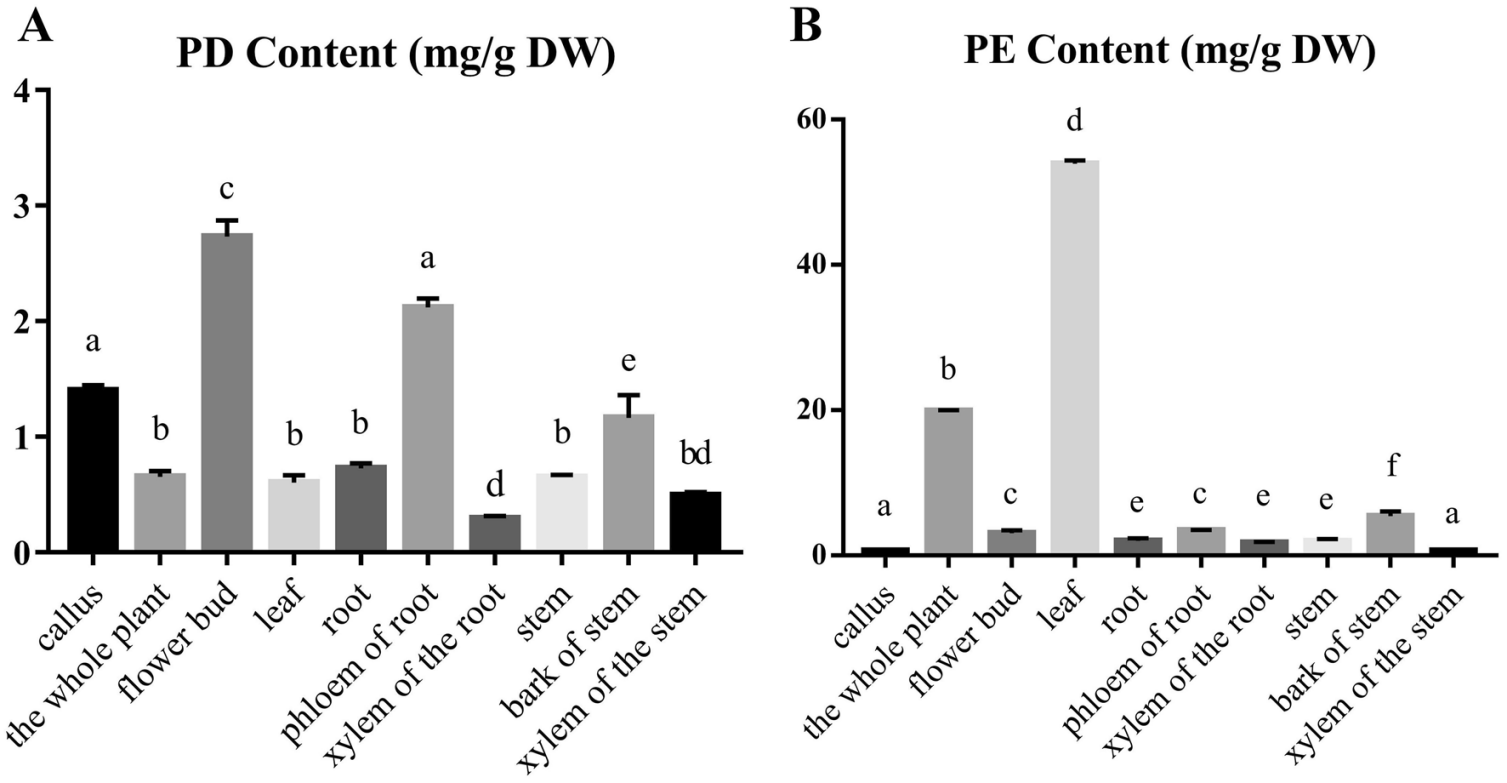
The contents of main triterpenoid saponins in callus and plant organs of *P*. *grandiflorus*. (**A**) Platycodin D (PD) content in different samples. (**B**) Platycoside E (PE) content in different samples. The X axis is the sample name and the Y axis is the content (mg/g DW), DW is dried weight. Bars with different letters within same histogram, represent significant difference at p ≤ 0.01.

Two kinds of specific platyopsis saponins PD and PE were identified by comparing the retention time^34^ and maximum uptake of the samples and standards at a wavelength of 210 nm by HPLC. The results showed that the concentrations of PD and PE are positively correlated with the area of the peak in the test range (R^2^ > 0.9999, R^2^ > 0.9946), and the content of each saponins was calculated from the peak area versus its own standard curve. The retention time and peak profile of PD and PE in standard and one sample are shown in Fig. 2. The content variance of PD and PE in callus and different organs in *P*. *grandiflorus* was shown in Supplementary Table S1 and Fig. 3.

ANOVA indicated that the distribution of PD content varied wildly among organs in plants of *P*. *grandiflorus* (Fig. 3A). It can be concluded that PD was most enriched in flower buds, followed by the roots, while PD content in leaves, stems and the whole plants is low. When it comes to the distribution of PD content in stems and roots, the contents in the bark and phloem are higher than that in the xylem. The results also showed that the content of PD in callus is higher than that of the whole plants, leaves, stems and roots, and it only lower than that in the flower buds. It can be inferred that PD could be obtained more efficiently from flowers and calli than from any other organs and tissues. Since the biomass of flower buds in the whole plants is very low, callus of *P*. *grandiflorus* may become an alternative material for PD production.

As for the distribution of PE content in *P*. *grandiflorus*, only the PE content in leaves is higher than that in the whole plant, while the PE content of flowers, roots and stems is much lower than that of the whole plant (Fig. 3B). It can be concluded from PE content distribution in each organ that the PE content of phloem and bark is higher than that of xylem which is consistent with the distribution of PD content distribution described above. The result also shows that the content of PE of calli is lower than that of all the organs in *P. grandiflorus*.

We found the content of PD in calli was higher than that in leaves which was sharply contrast to the highest PE and lower PD content in leaves. Experiments in vitro have shown that through enzymes catalysis, PE could convert to PD with higher pharmacological activities^18,19^. Therefore, we speculate the existence of putative enzymes converting PE to PD in *P*. *grandiflorus*. Based on the significant differences of PD and PE content between calli and leaves of *P*. *grandiflorus*, an RNA-Seq experiment was designed to identify the candidate genes.

### Total polysaccharide content variation among calli and plant organs

**Figure 4:**
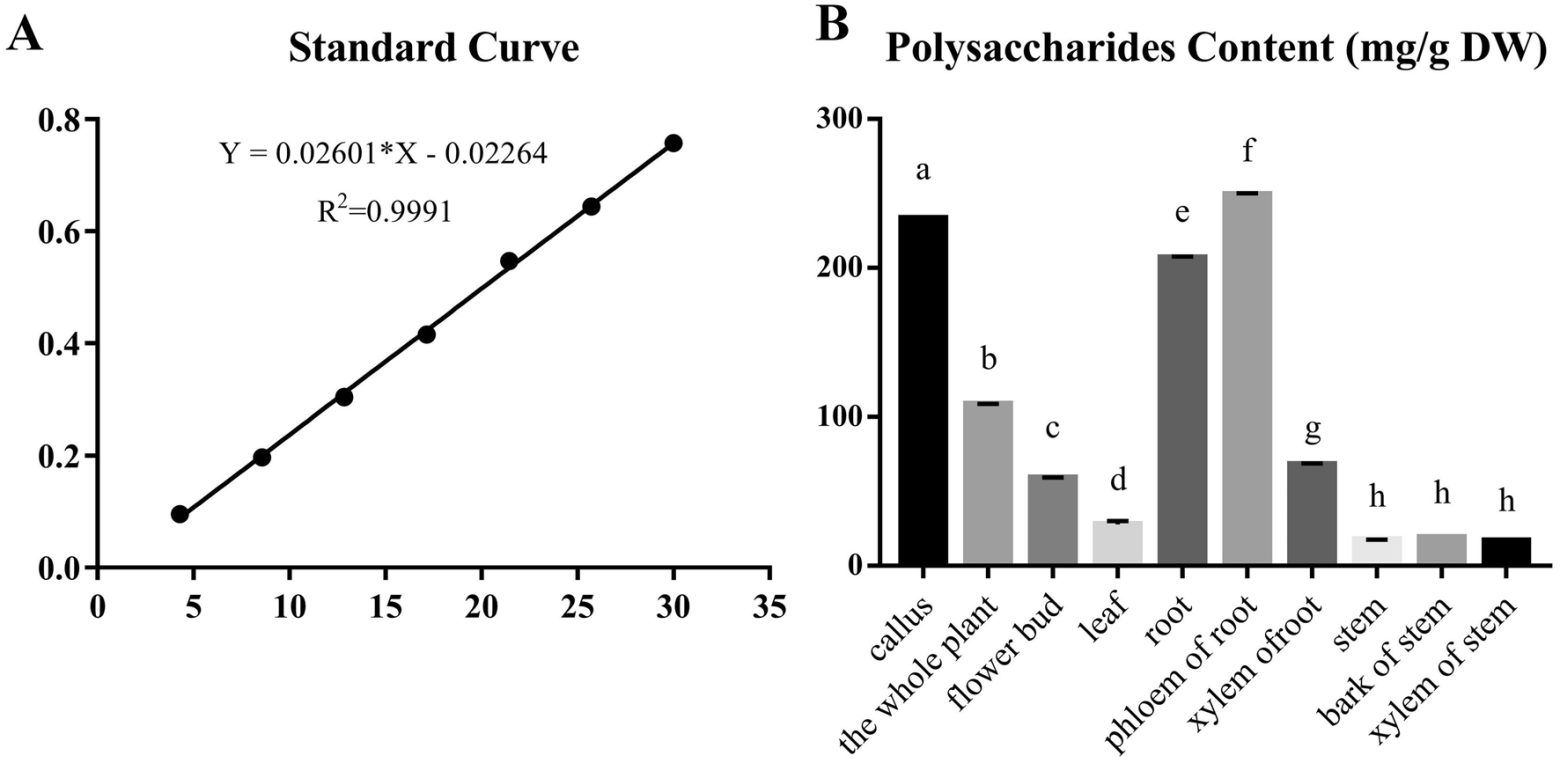
The contents of total polysaccharide among callus and different organ samples. (**A**) Polysaccharides content in different samples. The X axis is the sample name and the Y axis is the content (mg/g DW), and DW means dried weight. Bars with different letters within same histogram, represent significant difference at p ≤ 0.01. (**B**) Standard curve, R^2^ = 0.9991. The X axis is the standard concentration (μg/mL), and the Y axis is the absorbance value.

A standard curve was drawn from the absorbance value versus 7 gradient concentrations of standard glucose work solution with blank at 486 nm following chromogenic reaction (Fig. 4A). The regression equation of the standard curve is $\mathrm{Y }= 0.026\mathrm{X }- 0.0226$, R^2^ = 0.9991, showing that the quantification responds to linearity within the tested concentrations. The total polysaccharide content of callus and organs from the plant was detected by phenol-concentrated sulfuric acid method mentioned above (Fig. 4B) (Supplementary Table S1).

As for the distribution of polysaccharides in the *P*. *grandiflorus* also showed significant difference among samples by ANOVA, the content of polysaccharides of root is much higher than that of the whole plant which is consistent with the use of platycodi radix as medicinal part, which indicates that the roots of *P*. *grandiflorus* are an important place for the storage of polysaccharides. The results showed that the content of polysaccharides of the calli induced by this optimal medium is higher by comparing to the whole plants and organs in *P*. *grandiflorus*, implying callus might be a high-efficiency material and an important alternative for polysaccharides production than *P*. *grandiflorus* plants. At the same time, we also found that the polysaccharides content of root phloem was higher than that of the root xylem. This indicates that the root phloem is a more important place for storing polysaccharides. Moreover, we found that the content of polysaccharides of calli is much higher than that of leaves, which is in stark contrast to the PE content of callus and leaves.

### Sequencing and De novo assembly

A total of 39.44 Gb data were generated using the BGISEQ-500 platform by sequencing the established six libraries (leaf and callus each has three duplicates). The raw data were filtered by trimmomatic software (version 0.36, parameters are illuminaclip:2:30:10, leading:3, trailing:3, slidingwindow:4:15 minlen:50) to obtain clean reads by removing the reads containing connector, or unknown bases content more than 5%, or reads with low quality. The clean read numbers of each library were counted by SOAPnuke (version 1.4.0, parameters are -l 5 -q 0.5 -n 0.1). Trinity software was used to assemble clean reads, and then Tgicl software was used to cluster the transcripts to remove redundancy and obtain unigenes. Finally, 152,777 unigenes were obtained, which is higher than those (34,053 unigenes) obtained by Chunhua Ma et al^16^. The total length, average length, N50, and GC content were 228,154,936 bp, 1,493 bp, 2,514 bp, and 40.58%, respectively.

A single-copy ortholog database, BUSCO (https://busco.ezlab.org/) (Supplementary Figure S3), was used to evaluate the quality of the assembled transcripts. By comparing with conserved genes, the results showed the good integrity of the transcriptome assembly. A total of 80,826 coding sequences (N50 = 1,380) were identified, with a maximum length of 16,398 and a minimum length of 297 by TransDecoder software.

### Unigenes functional annotation and expression analysis of unigenes

Unigenes were compared to the seven major functional databases to annotate. There were 97,878 (NR: 64.07%), 82,833 (NT: 54.22%), 73,371 (SwissProt: 48.02%), 77,858 (KOG: 50.96%), 78,197 (KEGG: 51.18%), 73,988 (GO: 48.43%), and 73,302 (Pfam: 47.98%) unigenes received functional annotations. 80,826 CDS were detected using Transdecoder software. At the same time, 77,478 SSRs were distributed among 50,171 unigenes, and 3,153 unigenes encoding transcription factors were predicted (Supplementary Table S2: X1, X2 and X3 are the three independent replicates of leaves, X4, X5 and X6 are the three independent replicates of callus). The sequencing and analyzing data had been stored in website: https://www.ncbi.nlm.nih.gov/geo/query/acc.cgi?acc=GSE153777.

### Identification of candidate genes involved in triterpenoid saponin biosynthesis by expression level analysis

KEGG analysis was applied to gain insight into pathways of unigenes. A total of 22,842 unigenes are annotated in the KEGG database^20^. In order to discover the most important biological pathways, the KEGG metabolic pathways involved in genes are divided into 5 branches: cellular processes, environmental information processing, genetic information processing, metabolism, and organismal systems (Supplementary Table S2), including 19 subcategories (135 routes). Eight pathways (ko00906, ko00908, ko00909, ko00904, ko00903, ko00902, ko00900 and ko00905) of "Metabolism of terpenoids and polyketides" containing 864 unigenes were analyzed. These genes encoding enzymes for terpenoid synthesis that are mainly distributed in upstream of MEP and MVP, while some are distributed in downstream (Fig. 1) (Table 2).

**Table 2:** Putative key enzymes involved in the triterpenoid saponins biosynthesis pathway in *P. grandiflorus*.

Enzyme name	EC number
AACT (acetoacetyl-coenzyme A)	2.3.1.9
HMGS (hydroxymethylglutaryl-CoA synthase)	2.3.3.10
HMGR (3-hydroxy-3-methylglutaryl-coenzyme A reductase)	1.1.1.34
MVK (mevalonate kinase)	2.1.7.36
PMK (phosphomevalonate kinase)	2.7.4.2
MVD (mevalonate diphosphate decarboxylase)	4.1.1.33
GPPS (geranylgeranyl pyrophosphate synthase)	2.5.1.29
FPPS (Farnesyl-diphosphate synthase)	2.5.1.1, 2.5.1.10
SS (squalene synthase)	2.5.1.21
SE (squalene epoxidase)	1.14.14.17
β-AS (beta-amyrin synthase)	5.4.99.39
β-A28O (isolate CYP716A140 beta-amyrin 28-oxidase)	1.14.13.-
LS (lupeol synthase)	5.4.99.41
CAS (cycloartenol synthase)	5.4.99.8
IDI (isopentenyl-diphosphate Delta-isomerase)	5.3.3.2
DXS (1-deoxy-D-xylulose 5-phosphate synthase)	2.2.1.7
DXR (1-deoxy-D-xylulose 5-phosphate reductoisomerase)	1.1.1.267
CMS (2-C-methyl-D-erythritol 4-phosphate cytidylyltransferase (4-diphosphocytidyl-2C-methyl-D-erythritol synthase))	2.7.7.60
CMK (4-diphosphocytidyl-2-C-methyl-D-erythritol kinase)	2.7.1.148
MCS (2-C-methyl-D-erythritol 2,4-cyclodiphosphate synthase)	4.6.1.12
HDS ((E)-4-hydroxy-3-methylbut-2-enyl-diphosphate synthase)	1.17.7.1, 1.17.1.3
HDR (4-hydroxy-3-methylbut-2-enyl diphosphate reductase)	1.17.1.4
ACM59590 (β-Glucosidase)	3.2.1.21

**Figure 5:**
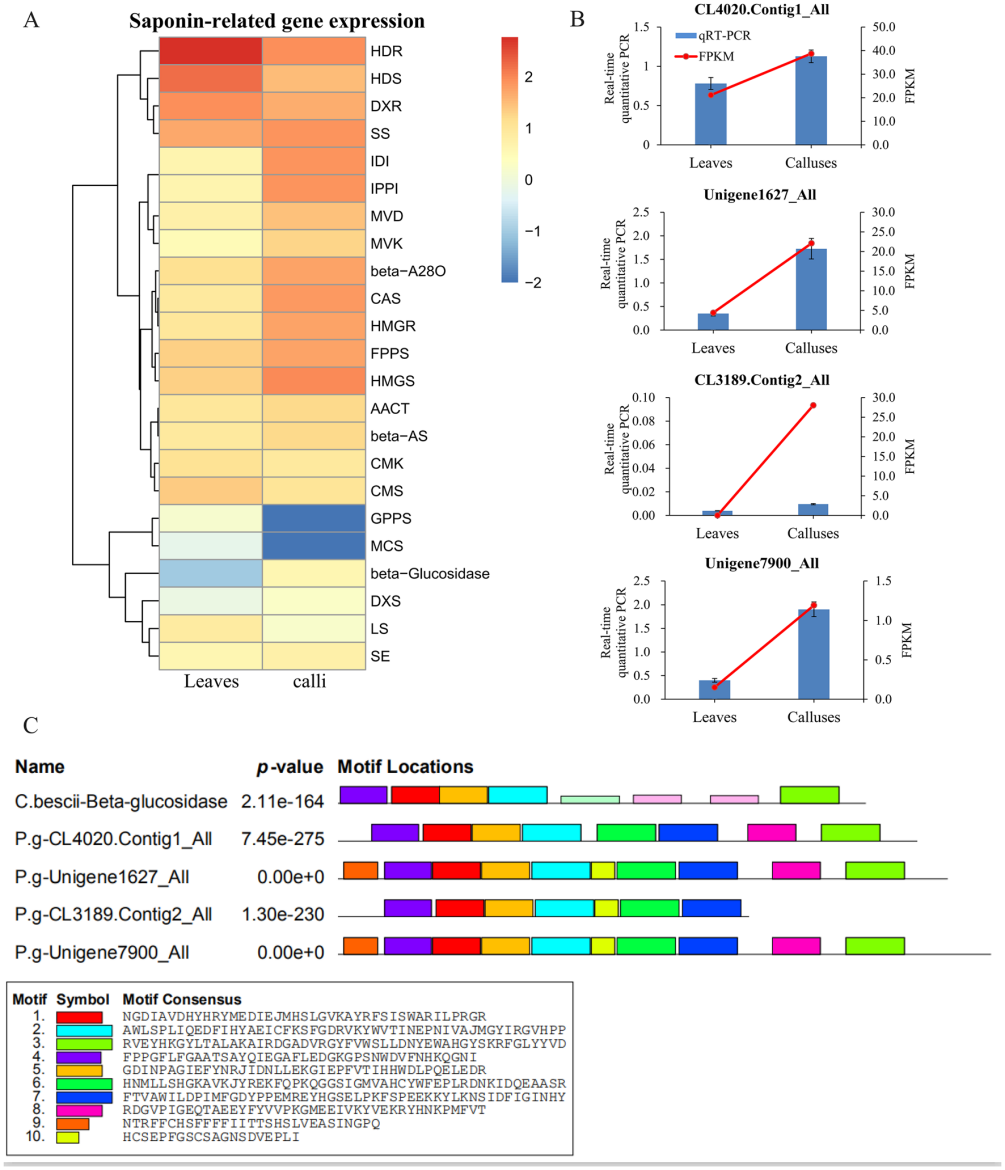
Expression level analysis of genes from triterpenoid saponins biosynthetic pathway in *P*. *grandiflorus* and conserve motif analysis of predictive β-glucosidase. (**A**) The expression levels of a single gene encoding an enzyme from each step of triterpenoid saponins biosynthetic pathway are shown. Red and green represent high and low expression levels, respectively. (**B**) Real-time quantitative PCR analysis of CL4020.Contig1_All, Unigene 1627_All, CL3189.Contig2_All and Unigene7900_All in leaves and calli. 18S rRNA as internal reference gene. Error bars indicate SD (n = 3). The blue bars represent the real-time quantitative PCR data and the red line represents the FPKM value from RNA-Seq. (**C**) Comparison of the conserve motifs among β-glucosidase from *C*. *bescii* and the amino acid sequences of putative orthologous unigenes from *P*. *grandiflorus*. Each block shows the position and strength of a motif site. The height of a block gives an indication of the significance of the site as taller blocks are more significant. The height is calculated to be proportional to the negative logarithm of the p-value of the site, truncated at the height for a p-value of 1e−10.

Expression level of putative genes encoding enzymes for triterpenoid saponins biosynthesis in *P. grandiflorus* between callus and leaf was measured by value of log2 (FC) of similar unigene from RNA-Seq data, and the similar unigene was obtained by aligning the amino acid sequences between putative genes and the unigene. Expression of genes encoding enzymes in the saponin biosynthetic pathway such as FPPS, HMGR, HMGS, IDI, MVD, MVK, SS, beta-A28O, beta-AS and beta-Glucosidase are up-regulated significantly (Fig. 5A). The expression level of a putative gene predicted to encode a de-glucosidase (ACM59590, β-Glucosidase) is consistent with the variance of PD and PE content, and two extracellular experiments indicate this enzyme may function as the β-Glucosidase, which showed that exogenous β-Glucosidase can catalyze the conversion of PE into PD through removing two glycosyl groups from PE in vitro^18,19^. From expression level analysis, 4 putative unigenes (CL4020.Contig1_All, Unigene 1627_All, CL3189.Contig2_All and Unigene7900_All) have high identity with the β-glucosidase from *Caldicellulosiruptor bescii* with amino acid sequence^18^. The result of real-time quantitative PCR indicated that the expression patterns of putative gene sequences encoding enzyme involved in converting PE to PD is consistent with the result from transcriptome analysis (Fig. 5B). Furthermore, to better select the putative sequences which might encode enzyme involved in converting PE to PD, the conserved motifs were analyzed using MEME (Fig. 5C). The result showed 3 unigenes (CL4020.Contig1_All, Unigene 1627_All and Unigene7900_All) were screened as candidate fragments of the target gene.

### Gene expression variance involved in polysaccharide biosynthesis

Furthermore, fifteen pathways (ko00010, ko00500, ko00620, ko00020, ko00051, ko00562, ko00052, ko00630, ko00030, ko00040, ko00053, ko00520, ko00640, ko00650 and ko00660) of “Carbohydrate metabolism” were analyzed, including 4,441 unigenes. There are 1,148 unigenes involved in starch and sucrose metabolism, and 765 unigenes involved in amino sugar and nucleotide sugar metabolism. Based on KEGG pathway analysis, we developed a model to infer the biosynthetic pathway of polysaccharides in *P. grandiflorus* (Supplementary Figure S4) (Table 3).

**Table 3:** Putative key enzymes involved in polysaccharide biosynthesis in *P. grandiflorus*.

Enzyme name	EC number
SUS (sucrose synthase)	2.4.1.13
INV (sucrose invertase)	3.2.1.26
HK (Hexokinase)	2.7.1.1
PGM (Phosphoglucomutase)	5.4.2.2
USP (UDP-sugar pyrophosphorylase)	2.7.7.64
UGP2 (UTP-glucose-1-phosphate Uridylyltransferase)	2.7.7.9
scrK (Fructokinase)	2.7.1.4
MPI (Mannose-6-phosphate isomerase)	5.3.1.8
UGDH (UDP-glucose 6-dehydrogenase)	1.1.1.22
UXS1 (UDP- glucuronate decarboxylase)	4.1.1.35
UXE (UDP-arabinose 4-epimerase)	5.1.3.5
RHM (UDP-glucose 4,6-dehydratase)	4.2.1.76
UER1(3,5-Epimerase-4-reductase)	5.1.3.-, 1.1.1.-
PMM (Phosphomannomutase)	5.4.2.8
GMPP (Mannose-1-phosphate Guanylyltransferase)	2.7.7.13
GMDS (GDP-mannose 4,6-dehydratase)	4.2.1.47
TSTA3 (GDP-_L_-fucose synthase)	1.1.1.271
GPI (Glucose-6-phosphate isomerase)	5.3.1.9
GALE (UDP-glucose 4-epimerase)	5.1.3.2
UGE (UDP-glucuronate 4-epimerase)	5.1.3.6
AXS (UDP-apiose/xylose synthase)	AXS
Amylosucrase (1,4-α-D-glucan 4-α-D-glucosyltransferase-glucan)	2.4.1.14

**Figure 6:**
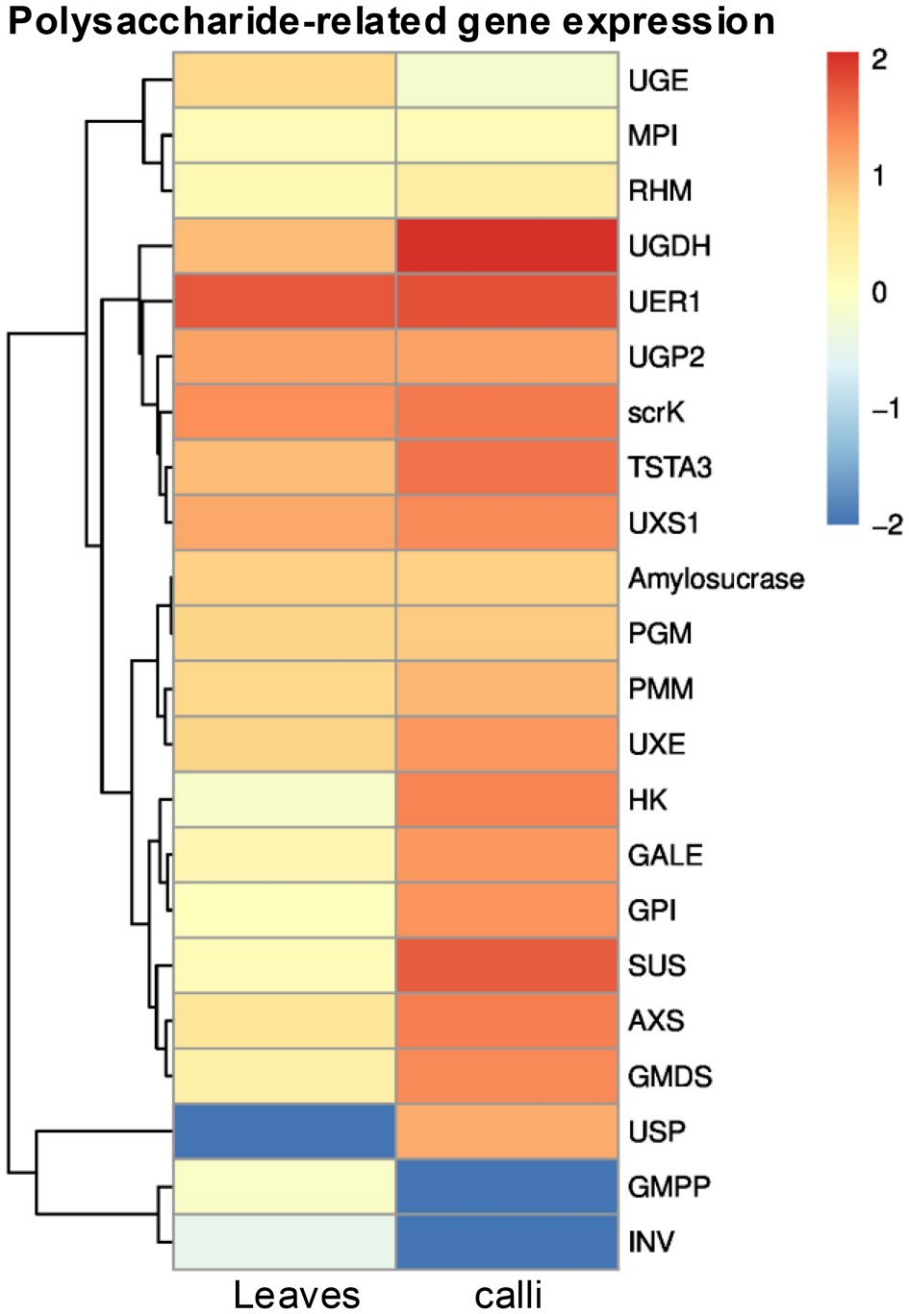
The expression levels of a single gene encoding an enzyme from each step of polysaccharides biosynthetic pathway in *P*. *grandiflorus* are shown. Red and green represent high and low expression levels, respectively.

The value of log2 (FC) was also used to analyze the expression of genes encoding enzymes for polysaccharide biosynthesis, and it showed that the expression levels of putative genes encoding AXS, GALE, GMDS, GPI, HK, PMM, SUS, TSTA3, UGDH, USP and UXE in the polysaccharide biosynthetic pathway were also upregulated significantly (Fig. 6). The result of gene expression quantity is consistent with the fact that the content of total polysaccharides in calli is higher than that of leaves.

### Detection of transcription factors

**Figure 7:**
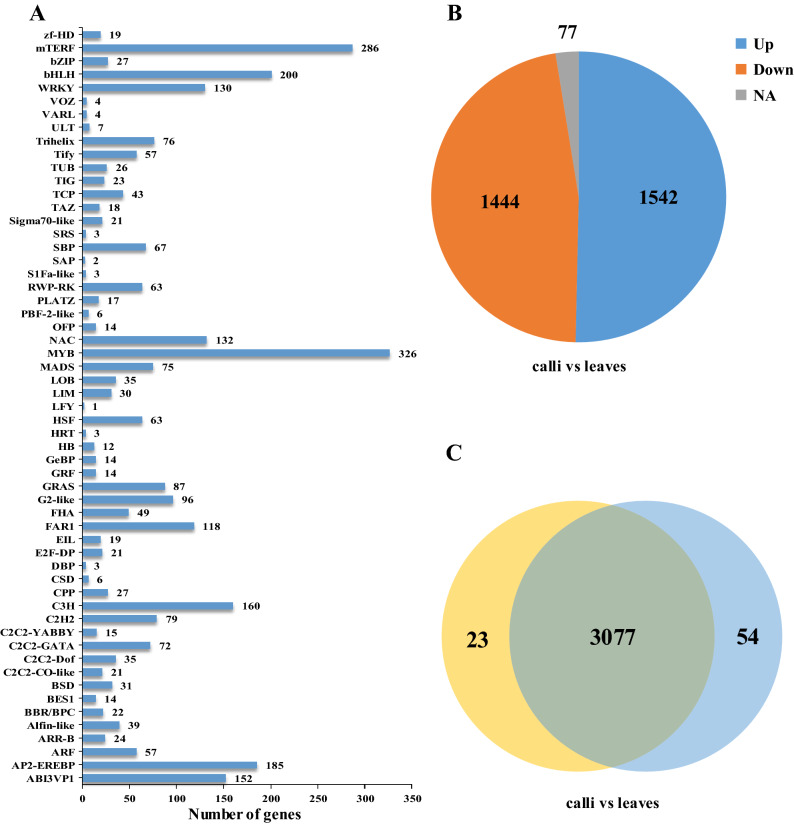
Transcription factors (TFs) expression analysis. (**A**) Classification of gene transcription factors’ family. (**B**) The expression level of TFs gene is different in calli vs. leaves, NA stands for expression only in calli or leaves. (**C**) Venn diagram of TFs gene expression in leaves and calli.

Transcription factors (TFs) can temporarily and spatially regulate the activity of target genes, and play a key role in plant development and response to the external environment^36–38^. A total of 3,153 candidate TFs were identified in the *P. grandiflorus* transcriptome database and classified into 57 different TF families (Fig. 7A), among these families that MYB family (326 unigenes) accounted for the largest proportion of TF families, followed by mTERF (286 unigenes), bHLH (200 unigenes), AP2-EREBP (185 unigenes), C3H (160 unigenes), ABI3VP1 (152 unigenes), NAC (132 unigenes), WRKY (130 unigenes), and FAR1 (118 unigenes). In these target TFs 1,542 were up-regulated and 1,444 were down-regulated in the calli versus leaves (Fig. 7B). We found that 54 candidate genes among transcription factors are expressed only in leaves, and 23 are expressed only in calli (Fig. 7C) (Supplementary Table S3).

By KEGG pathway analysis it can be concluded that total 11 (4 Trihelix and 7 FHA) and 15 (3 bHLH, 6 C2H2, 3 Trihelix, 1 G2-like, 1 FHA and 1 VARL) TFs may relate to the biosynthesis of saponins and polysaccharides, respectively (Supplementary Table S4). It also found a Trihelix TF gene (Unigene26781_All) is co-expressed with the putative de-glucosylation enzyme gene (β-Glucosidase).

## Discussion

Plant tissue culture is not only a method for plant rapid propagation, but also an ideal way for plant improvement, germplasm preservation and production of useful compounds. Studies have shown that compounds can be quickly obtained from callus of *Citrus junos* Siebold ex. Tanaka^39^ and *Ocimum basilicum* L.^18,19^. It was reported that there was high content of pentacyclic triterpenoids with anti-inflammatory and antinociceptive activities in callus of *Chaenomeles japonica* (Thunb.) Lindl. ex Spach^40^. So far, there’s no specific protocol for the callus induction of *P. grandiflorus*. The best callus induction plan of *P*. *grandiflorus* was screened out through experiments in this article, which filled the research gap, and provided a basis for the development and utilization of *P*. *grandiflorus* callus.

The contents of PE and PD in the phloem of *P*. *grandiflorus* is higher than that in the xylem like some other secondary compounds^41,42^, indicating that the phloem of *P*. *grandiflorus* may have the function to transport and store PD and PE. In addition, we found that the content of PE is the highest in leaves, while that in callus was lower than that in other organs. So, obtaining PE from leaves of *P*. *grandiflorus* is probably the best choice*.*

Studies have shown that PE converted to PD by removing two molecules of glycosyl group under the action of deglycosylated enzymes^18,19^. Since callus has much higher content of polysaccharides and PD, and lower content of PE than leaves, then we highly speculate that the active glycosyltransferase in the callus will catalyze the conversion of PE into PD, and the glucose groups were released to participate in the biosynthesis of polysaccharides. This process has a positive effect on the accumulation of polysaccharides in callus.

The number of unigenes is 152,777 of us versus 34,053 of Ma’s^16^. The average length and N50 values of genes in this study are also higher than those in Ma’s results (with the total average length of 936 bp and N50 of 1,661 bp)^16^, and the differences are mainly due to the different materials and sequencing platform used for RNA-Seq, and different sequencing depth. It was reported that endophytic bacteria are involved in secondary metabolite biosynthesis, which could be isolated from the interior of *P. grandiflorum*^43^, which means there might be genes in the endophytic bacteria encoding key enzymes catalyzing the formation of secondary metabolites^44^. So, the calli and leaves of *P. grandiflorum* are used as materials for RNA-Seq to avoid the residual RNA from endophytes. Transcriptome analysis were quite important methods to identify new genes in triterpenoid saponin biosynthetic pathway in *P. grandiflorum*. Besides, the difference in plant materials between Ma^16^ et al. and us. Many key enzymes involved in triterpenoid saponin biosynthesis were discovered in both studies, including HMGS, HMGR, FPPS, SS, SE, et al. However, UGTs cloned in Ma’s study catalyze the glucosylation; in our work, 3 unigenes or orthologous sequences (CL4020.Contig1_All, Unigene 1627_All and Unigene7900_All) were screened as candidate gene which can catalyze degradation of glycosyl group and convert PE to PD. Our research could supply more transcriptome data, and it is the first time to identify the candidate genes which that converts PE to PD in *P. grandifloras.*

Previous studies have shown TFs, such as the bHLH transcription factor AabHLH1 and AaMYC2 in *Artemisia annua* L., have effects on the primary and secondary metabolites of plants, that can effectively regulate the biosynthesis of artemisinin^45,46^, and the study has shown that the Trihelix family transcription factor BdTHX1 likely plays an important role in mixed-linkage glucan biosynthesis and restructuring by regulating the expression polysaccharide synthase related genes^47^. It was reported that Trihelix TF, responding to light signals, regulates the expression of downstream gene by calcium-dependent phosphorylation and dephosphorylation^48^. The expression level of a Trihelix TF gene (Unigene26781_All) is co-expressed with the putative deglucosylation enzyme gene (β-Glucosidase) in this study, which demonstrated that this Trihelix TF might sense the light signal, and influence the expression of the putative gene encoding β-Glucosidase, then modify and regulate the conversion of PE to PD. The discoveries of these TFs may have great potential value and broad application prospects in studying the regulation of terpenoid saponin and polysaccharide bio-synthesis in *P*. *grandiflorus*.

## Conclusions

A best protocol for calli induction of *P. grandiflorus* is that use the stem with nodes as explants, and take MS + NAA 1.0 mg/L + 6-BA 0.5 mg/L as the formula. We found that the content of PD and polysaccharide in callus is higher than that in the plant of *P*. *grandiflorus*, as well higher than that in the root which is traditional medicinal parts. Study also showed that PD content of calli is higher than that of leaves, which is in sharp contrast to PE content.

We performed comprehensive RNA-Seq analysis on the leaves and calli of *P. grandiflorus*, and were able to find the expression of many genes involved in triterpenoid saponins and polysaccharides which were co-expression with the content of corresponding metabolites. Three putative unigenes with high amino acid sequence identity were screened as orthologous sequences of candidate β-Glucosidase gene converting PE to PD, which is helpful to deeply understand the biosynthesis mechanism of triterpene saponins in *P*. *grandiflorus* at the molecular level. A total of 11 TFs were involved in regulating the biosynthesis of saponins, and 15 TFs were involved in carbohydrate metabolism were obtained.

In summary, our work may greatly help to promote the molecular biology research and improve the large-scale production of triterpene saponins and polysaccharides in *P. grandiflorus*.

## Methods

### Plant materials

Plants used in this experiment were identified as *Platycodon grandiflora* (Jacq.) A. DC.by expert who is major in plant identification, which were grown at 25 °C during day and 23 °C at night in the green house of Anhui University of Chinese Medicine, Anhui, China. The seedlings of *P*. *grandiflorus* were purchased from Bozhou Market of Traditional Chinese Medicine, Anhui Province, China. Specimen of *P*. *grandiflorus* in this study was deposited in the Herbarium of Anhui University of Chinese Medicine and the depository No. is 20,200,705. The explants used for callus induction and the materials used for metabolites determination and RNA-Seq are all from the same plantlets. Each sample in this study has three independent biological duplicates.

### Chemicals

Standard compounds of platycodin D (PD, > 98% purity) and platycodin E (PE, > 98% purity) were purchased from Chengdu Desite Biotech Co., Ltd. and Chengdu Push Bio-technology CO., Ltd., respectively. CTAB-PBIOZOL reagent was purchased from Bioflux. Acetonitrile and Methanol (HPLC grade) were purchased from Oceanpak. HPLC-grade water was prepared using laboratory water purification system from Pall Filter Co., Ltd. (Beijing, China). Plant growth regulators of 6-BA (6-Benzylaminopurine) and α-NAA (α-Naphthylacetic acid) were purchased from Beijing Solarbio Technology Co., Ltd. Chemicals for plant tissue culture were bought from Sinopharm group, and all other chemicals were of analytical grade.

### Calli induction of *Platycodon grandifloras*

In order to screen the optimal processes of inducing calli of *P*. *grandiflorus* included optimum explants, basic medium and plant growth regulators combination, a L_9_(3^4^) orthogonal experiment was carried out based on the documents and our previous study regardless of the interactions among factors (Supplementary Table S5). The best one was selected from basic media (Factor A) of B5, MS and WPM for *P*. *grandiflorus* calli induction. Leaves, stems with nodes and stems without nodes were used as explants (B), which are in the same batch with the materials used for content detection and RNA-Seq analysis. In order to select the optimal combination for calli induction of *P*. *grandiflorus*, plant growth regulator combinations with different concentrations of auxin and cytokinin were designed according to relevant literature. NAA (C) and 6-BA (D) were divided into three concentrations of 0.1 mg/L, 0.2 mg/L and 1.0 mg/L and 0.5 mg/L, 1.0 mg/L and 2.0 mg/L, respectively.

Basic medium B5 (Gamborg B5 Medium), MS (Murashige and Skoog Medium), WPM (Lloyd & McCown Woody Plant Basal Medium)^49^ stock solutions were prepared. The full media formulas were prepared according to the different proportions of components, and different concentrations of plant growth regulators. Finally, adjusted the pH of media to 5.6 ~ 6.0, followed by the addition of sucrose (30 g/L) and agar (7 g/L), then the media was sterilized by autoclaving at 121 °C for 30 min.

Explants of *P. grandiflorus* were rinsed and surface-sterilized, and rinsed 3–5 times with sterile distilled water. The sterile explants were dried, leaves were cut into 0.5 cm^2^ in size, and the stems were cut into pieces about 1 cm in length, then they were inoculated onto the media. Each group of explants were inoculated in 20 bottles with 3 pieces per bottle. The culture conditions were as follows: the photosynthetic photon flux density was 30 μmol/m^2^/s for 12 h/d, the culture temperature was (25 ± 1) °C, and the culture lasted for 50 d.

### Extraction of triterpenoid saponin D and E

Jaeyoung Kwon et al.^2^ found that PD degraded in the minimum at 40 °C in drying process, and suitable for detection. In this paper, all the organs of wild plants and tissue culture materials of *P*. *grandiflorus* were dried at 40 °C to constant weight and pulverized by a ball mill. Then accurately weighed the sample powders (each sample 0.5 g) and transferred them to a 50 mL of centrifuge tubes. After adding of 25 mL methanol, each sample solution was adjusted to the same weight and recorded the final weight followed by ultrasonic extraction lasting for 50 min. And the loss of solution occurring during ultrasonic extraction was compensated for by adding a certain amount of 100% methanol to the same weight. Finally, 20 mL of continuous filtrated to 2 mL for further use.

### HPLC quantitative analysis of saponin D and E from calli and organs of plants

All analyses of PD and PE contents were performed on an Agilent Series 1260 system (Agilent Technologies, Germany). Topsil C18 HPLC column (4.6 mm × 250 mm, 5 µm particle size) was used for chromatography. Elution was carried out using (A) water and (B) acetonitrile as a mobile phase. The ratio of A to B is 71: 29. The flow rate was 1.0 mL/min, the sample injection volume was 5 µL, and the column temperature was 30 °C. The Detection wavelength was 210 nm.

### Extraction and determination of total polysaccharide content

Polysaccharides extraction from *P*. *grandiflorus* and determination were performed by the methods reported previously with a few modifications^50^. All the samples were dried at 40 °C to constant weight and milled to powder. Accurately weighed samples (0.5 g each sample), and added 50 mL distilled water to mix evenly. Subsequently, weighed the mixture solution and allowed it to stand for 30 min, and then refluxed and extracted in a boiling water bath for 1 h. When the solution was cold, the loss of the solutions was compensated by adding distilled water, then shook and filtered it. Precisely took 10 mL of the filtrate and evaporated it, added 2 mL distilled water to dissolve the evaporated sample and then mixed the solution with 10 mL of absolute ethanol to dissolve for 24 h. After centrifugation at 4000 g for 10 min, removed the supernatant and washed the precipitate 3 times with 85% ethanol. Then the precipitate was dissolved in 50 mL of water and shook intermittently and solution for test was obtained. 1 mL of each raw material was used to determine the total polysaccharide content by the improved sulfuric acid phenol method.

The standard glucose solution (0.3 mg/mL) was prepared as follows: glucose of 7.5 mg in chromatographic grade was dissolved in distilled water and diluted to a constant volume of 25 mL. Gradient solutions were prepared by transferring 0.1, 0.2, 0.3, 0.4, 0.5, 0.6, 0.7 mL of standard glucose solution to the test tubes and added distilled water to a constant volume of 1 mL. Phenol sulfate method was used to determine and draw the standard curve, and the absorbance were measured at 486 nm using spectrophotometer.

### RNA extraction

Total RNA was purified by CTAB-PBIOZOL reagent as extraction solution from leaves and calli of *P*. *grandiflorus*. Around 80 mg samples were ground into powder in liquid nitrogen and transferred to tubes containing 1.5 mL of preheated 65 °C CTAB-pBIOZOL reagents. The samples were incubated by Solarbio mixer for 15 min at 65 °C to make the nucleoprotein complexes in the samples completely dissolved. After centrifugation at 12,000 g at 4 °C for 5 min, the supernatants were mixed with 400 µL of chloroform per 1.5 mL of extraction solution and centrifuged at 12,000 g for 10 min at 4 °C. After centrifuge, the supernatants were transferred to a new 2.0 mL test tubes containing 700 µL acidic phenol and 200 uL chloroform followed by centrifuging at 12,000 g for 10 min at 4 °C. Subsequently, the aqueous phase of each sample was mixed with an equal volume of chloroform before centrifuging at 12,000 g for 10 min at 4 °C. The supernatants were mixed with an equal volume of isopropyl alcohol and placed in − 20 °C for 2 h to precipitate, then centrifuged at 12,000 g for 20 min at 4 °C, and the supernatants were removed. After washing twice with 1 mL of 75% ethanol, they were dried in the bio-safety cabinet, and dissolved with 50 µL of sterilized DEPC-treated water. Finally, total RNA of each samples was qualified and quantified by a Nano Drop and Agilent 2100 bioanalyzer (Thermo Fisher Scientific, MA, USA).

### Construction of cDNA and RNA-Seq libraries and sequencing

Oligo(dT) attached magnetic beads were used to purified mRNA from leaves and calli of *P*. *grandiflorus* in three replicates by eliminating rRNA and tRNA in the total RNA. Purified mRNA was fragmented into small pieces with fragment buffer at appropriate temperature. Then, First-strand cDNA was generated by random hexamer-primed reverse transcription, followed by a second-strand cDNA synthesis. After that, RNA Index Adapters and A-Tailing Mix were added by incubating to end repair. The fragments of cDNA obtained from previous steps were amplified by PCR, and the PCR products were purified by Ampure XP Beads, and dissolved in EB solution. The products were validated on the Agilent Technologies 2100 bioanalyzer for quality control. The double stranded PCR products obtained from previous steps were denatured by heating and circularized by the splint oligo sequence to get the final library, and the single stranded circular DNA (ssCir DNA) was formatted as the final library.

The constructional final six libraries were further amplified with phi29 to make DNA nanoball (DNB), each molecular of which had more than 300 copies, DNBs were loaded into the patterned nano-array and paired-end of 150 bp base reads were generated on BGIseq 500 platform (BGI-Shenzhen, China).

### De novo transcriptome assembly and unigenes annotation

After sequencing, raw data were obtained, and low-quality, joint contamination and unknown base N were filtered by software SOAPnuke (version 1.4.0) to generate clean data. The filtered data which is called clean reads were de novo transcriptome assembled using Trinity software (version 2.0.6). The acquired full-length transcripts for alternatively splicing isoforms were obtained by splicing transcripts corresponding to paralogous genes, then the redundant transcripts were removed by TGICL (version 2.06, parameters: − 1 30 –v 35) to acquire non-redundant sequences which called unigenes. TransDecoder software (Version 3.0.1, parameters: default) was used to identify candidate coding regions in unigene^51^.

The assembled unigenes were subjected to databases as KEGG (Kyoto Encyclopedia of Genes and Genomes), NT (NCBI non-redundant nucleotide sequence), NR (NCBI non-redundant protein sequences), SwissProt (a manually annotated and reviewed protein sequence database) and KOG (Clusters of Eukaryotic Orthologous Groups) by Blast software (version 2.2.23)^52^ with default parameters (under a threshold E-value ≤ 10–5) to get the functional annotations. Ultimately, GO (Gene Ontology) annotations and functional classifications were obtained using Blast2GO program (version 2.5.0, E-value ≤ 10–6)^53^ based on NR annotations.

### Identification of differentially expressed genes (DEGs)

All clean reads from all samples were aligned to unigene sets using Bowtie2 (V2.2.5) with default settings^54^. The expression level of genes were calculated by RSEM (v1.2.8) ^55^ and normalized to fragments per kilobase of transcript per million (FPKM). DEseq2 (v1.4.5)^56^ was used to detect DEGs (Different expressed genes) with Q value (adjust P value) < 0.001, Unigenes showing differential expression between two issue types (leaf vs callus) at fold change ≥ 2 or ≤ -2, and a false discovery rate (FDR) ≤ 0.001 was identified as DEGs using the Posisson distribution method^57^. The identified DEGs were subsequently carried into GO and KEGG enrichment with Phyper in R package by Q value ≤ 0.05 as default. The heatmap was drawn by pheatmap (v1.0.8)^56^ according to the gene expression in different samples.

### Analysis of genes involved in saponin and polysaccharide biosynthesis.

According to the KEGG annotation results and official classification, we classified the differential genes in biological pathways, and used the phyper function in R software to perform enrichment analysis, calculated the p-value, and then performed FDR correction on the p-value. Generally, functions with Q-value ≤ 0.05 are considered significant enrichment (https://en.wikipedia.org/wiki/Hypergeometric_distribution). The logarithm of normalization of average FPKM and 0.01 (avoiding the value of FPKM is zero) was used to measure the expression level of each gene in leaf and callus. The comparison of conserve motif between putative candidate gene and the target gene was performed based on the results of MEME (http://meme-suite.org/tools/meme) analysis.

### Real-time quantitative PCR analysis

In order to verify RNA-Seq data, real-time quantitative PCR analysis was performed. Collected leaves and calli samples (each in three independent biological duplicates), which is same to RNA-Seq, and promptly frozen in liquid nitrogen and stored at − 80 °C. Total RNA was extracted according to the previous RNA extraction method. 0.5 μg of RNA was used to synthesize the cDNA using FastKing RT Kit (Tiangen, China). Real-time quantitative PCR analysis was conducted by using SuperReal PreMix Plus (Tiangen, China) on the LightCycle480 machine (Roche, Switzerland). The primer sequences of genes were designed by Primer Premier (version 5.0) (Supplementary Table S6). Housekeeping gene 18sRNA of *P*. *grandiflorus* was used as internal reference gene. The data from real-time quantitative PCR was statistical analyzed by the 2^−ΔΔCt^ approach.

### Analysis of transcription factors

ORFs (open reading frames) of unigenes were mapped to TF protein domain in PlnTFDB (plant TF database) based on BlastTX (E-value ≤ 1e−5) using Hmmsearch method^53^. High-expressing IFs corresponding to the saponin and polysaccharide were selected by comparing the value of log2(FC) of each IF in samples.

### Statistical analysis

All experiments in this study were performed with three independent biological duplicates including RNA-Seq and HPLC analysis, and the data are expressed as the mean ± standard deviation (SD) of three duplicates. The tissue culture data were analyzed by orthogonal experimental analysis, and the statistical differences of saponin D, E and polysaccharide contents among different samples, and the significance of difference from different factors on calli induction were analyzed using analysis of one-way and two-way variance (ANOVA) by SPSS software package (version 17.0), respectively. Values at P ≤ 0.01 were considered statistically significant difference.

## Supplementary Information

Supplementary Information 1.

Supplementary Information 2.

Supplementary Information 3.

Supplementary Information 4.

Supplementary Information 5.

Supplementary Information 6.

Supplementary Information 7.

Supplementary Information 8.

Supplementary Information 9.

Supplementary Information 10.

Supplementary Information 11.

## Data Availability

The datasets generated and analyzed during the current study are available in the [GEO] repository, [https://www.ncbi.nlm.nih.gov/geo/query/acc.cgi?acc=GSE153777].

## Abbreviations

AACT: Acetoacetyl-coenzyme A
6-BA: 6-Benzylaminopurine
Amylosucrase: 1,4-α-D-glucan-4-α-D-glucosyltransferase-glucan
AXS: UDP-apiose/xylose synthase
B5: Gamborg B5 Medium
CAS: Cycloartenol synthase
CMK: 4-Diphosphocytidyl-2-C-methyl-D-erythritol kinase
CMS: 2-C-methyl-D-erythritol 4-phosphate cytidylyltransferase (4-diphosphocytidyl-2C-methyl-D-erythritol synthase)
DXR: 1-Deoxy-D-xylulose 5-phosphate reductoisomerase
DXS: 1-Deoxy-D-xylulose 5-phosphate synthase
FPPS: Farnesyl-diphosphate synthase
GALE: UDP-glucose 4-epimerase
GMDS: GDP-mannose-4,6-dehydratase
GMPP: Mannose-1-phosphate Guanylyltransferase
GPI: Glucose-6-phosphate isomerase
GPPS: Geranylgeranyl pyrophosphate synthase
HDR: 4-Hydroxy-3-methylbut-2-enyl diphosphate reductase
HDS: (E)-4-hydroxy-3-methylbut-2-enyl-diphosphate synthase
HK: Hexokinase
HMGR: 3-Hydroxy-3-methylglutaryl-coenzyme A reductase
HMGS: Hydroxymethyl glutaryl-CoA synthase
IDI: Sopentenyl-diphosphate Delta-isomerase
INV: Isopentenyl-diphosphate Delta-isomerase
LS: Lupeol synthase
MCS: 2-C-methyl-D-erythritol 2,4-cyclodiphosphate synthase
MEP/DOXP: Non-mevalonate pathway
MPI: Mannose-6-phosphate isomerase
MS: Murashige & Skoog Medium
MVA: Mevalonic acid pathway
MVD: Mevalonate diphosphate decarboxylase
MVK: Mevalonate kinase
PD: Platycodin D
PE: Platycoside E
PGM: Phosphoglucomutase
PGPS: *Platycodon grandiflorus* Polysaccharides
PMK: Phosphomevalonate kinase
PMM: Phosphomevalonate kinase
RHM: UDP-glucose-4,6-dehydratase
SacA: β-Fructofuranosidase
ScrA: Sucrose-specific enzyme II
ScrK: Fructokinase
SE: Squalene epoxidase
SS: Squalene synthase
SUS: Sucrose synthase
TSTA3: GDP-L-fucose synthase
UER1: 3,5-Epimerase-4-reductase
UGDH: UDP-glucose-6-dehydrogenase
UGE: UDP-glucuronate 4-epimerase
UGE: UDP-glucuronate 4-epimerase
UGP2: UTP-glucose-1-phosphate Uridylyltransferase
USP: UDP-sugar pyrophosphorylase
UXE: UDP-arabinose-4-epimerase
UXS1: UDP-glucuronate decarboxylase
WPM: Lloyd & McCown Woody Plant Basal Medium
α-NAA: α-Naphthylacetic acid
β-A28O: Isolate CYP716A140 beta-amyrin 28-oxidase
β-AS: Beta-amyrin synthase

## Guidelines and legislation

The authors declare that the experimental research and field studies on plants, including the collection of plant material, complied with our institutional, national, and international guidelines and legislation.
