# Supplementary material for: A candidate gene identified in converting platycoside E to platycodin D from Platycodon grandiflorus by transcriptome and main metabolites analysis
Source: Sci Rep. 2021 May 7;11:9810. doi: 10.1038/s41598-021-89294-1 (PMC8105318; doi:10.1038/s41598-021-89294-1)
Supplement: Supplementary file 1 — Supplementary Information 1. [file 41598_2021_89294_MOESM1_ESM.docx]

**Supplementary information**

**Supplementary Figure S1.** Predicted polysaccharide biosynthetic pathways in *P. grandiflorus*. Arrows with solid lines represent the identified enzymatic reactions, and arrows with dashed lines represent multiple enzymatic reactions through multiple steps.

**Supplementary Figure S2.** Calli induced from *P. grandiflorus* explants.

**Supplementary Figure S3.** The quality of the assembled transcripts was evaluated using a single-copy orthologous database, BUSCO.

**Supplementary Figure S4.** KEGG pathway annotated classification results of differential expression genes (DEGs).

**Supplementary Table S1.** The contents of platycoside E, platycodin D and polysaccharides among samples.

**Supplementary Table S2.** Table of unigenes information from RNA-Seq.

**Supplementary Table S3.** Table of differential expression transcription factors.

**Supplementary Table S4.** Differential expression of transcription factors involved in the metabolism of saponins and polysaccharides.

**Supplementary Table S5.** Design of the header for orthogonal experiment of induced callus.

**Supplementary Table S6.** The primer sequences and results of real-time quantitative PCR for the putative gene sequences.
