# Supplementary figures and images for: A candidate gene identified in converting platycoside E to platycodin D from Platycodon grandiflorus by transcriptome and main metabolites analysis
Source: Sci Rep. 2021 May 7;11:9810. doi: 10.1038/s41598-021-89294-1 (PMC8105318; doi:10.1038/s41598-021-89294-1)

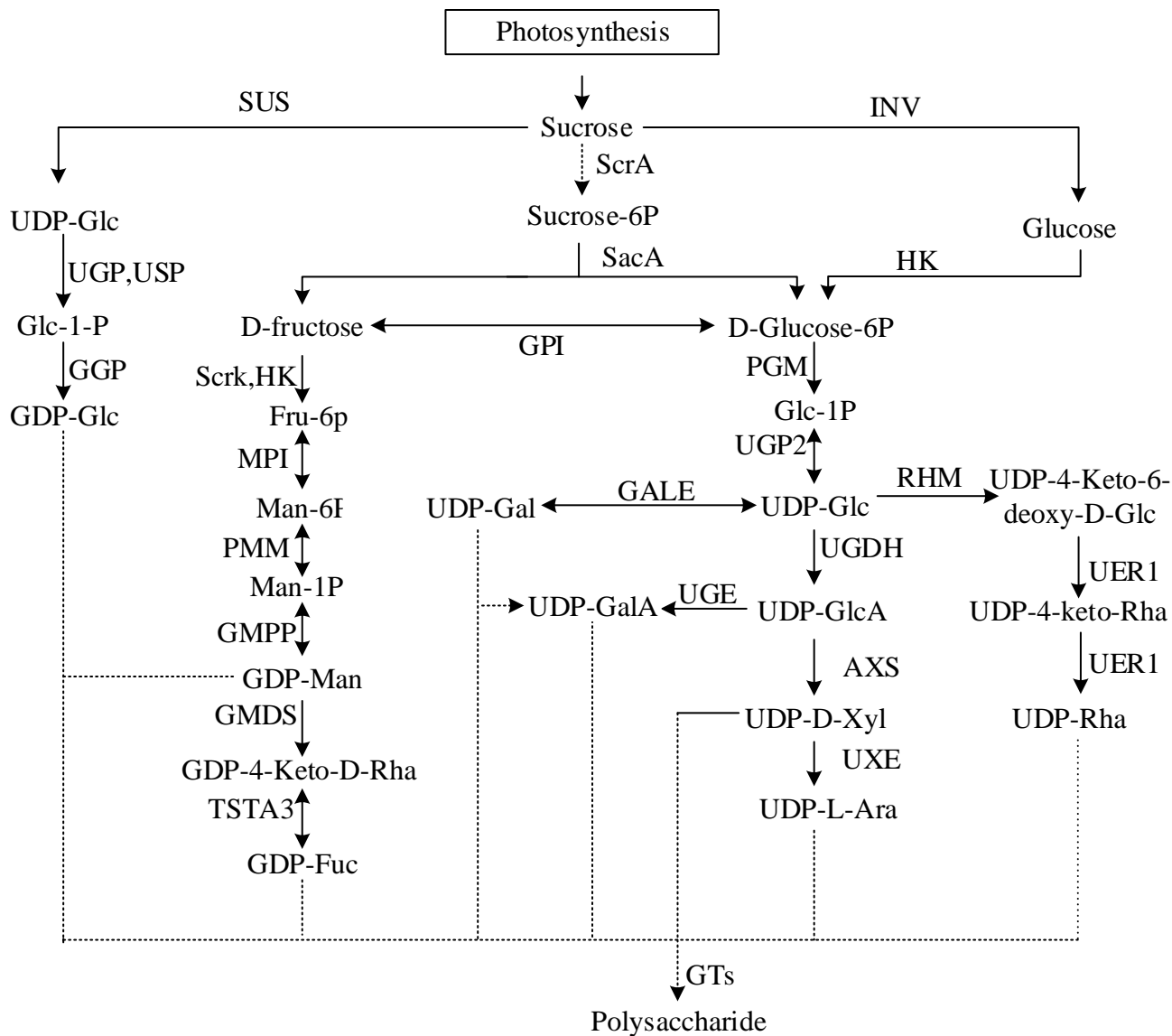

Supplement: Supplementary file 2 — Supplementary Information 2. [file 41598_2021_89294_MOESM2_ESM.pdf]

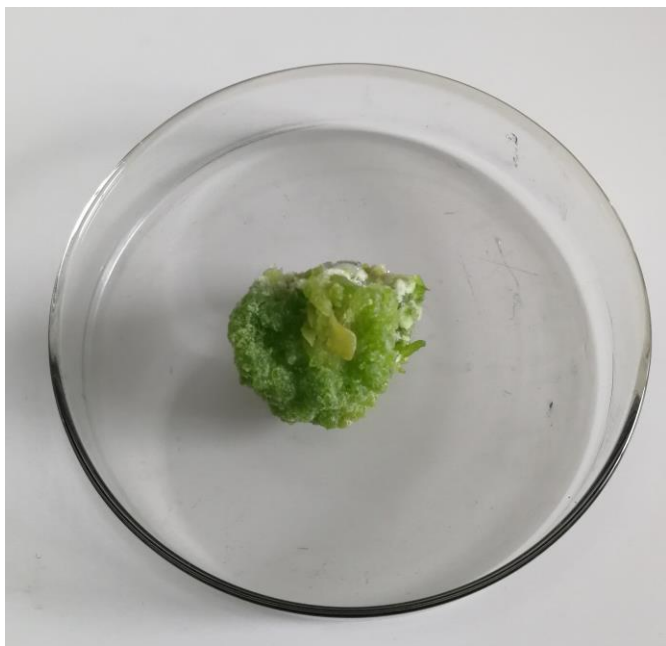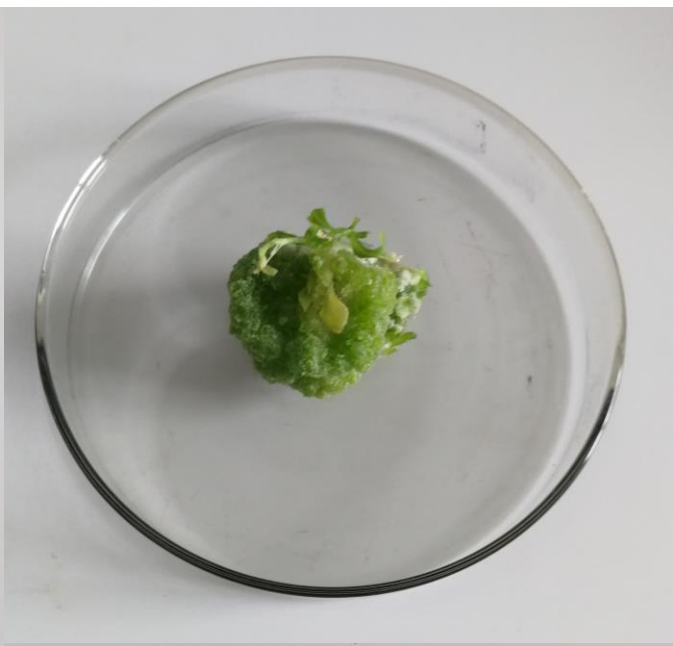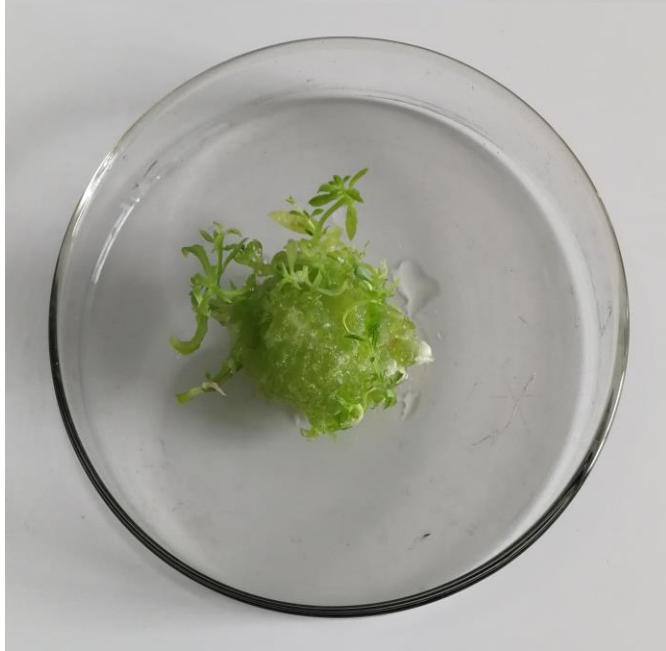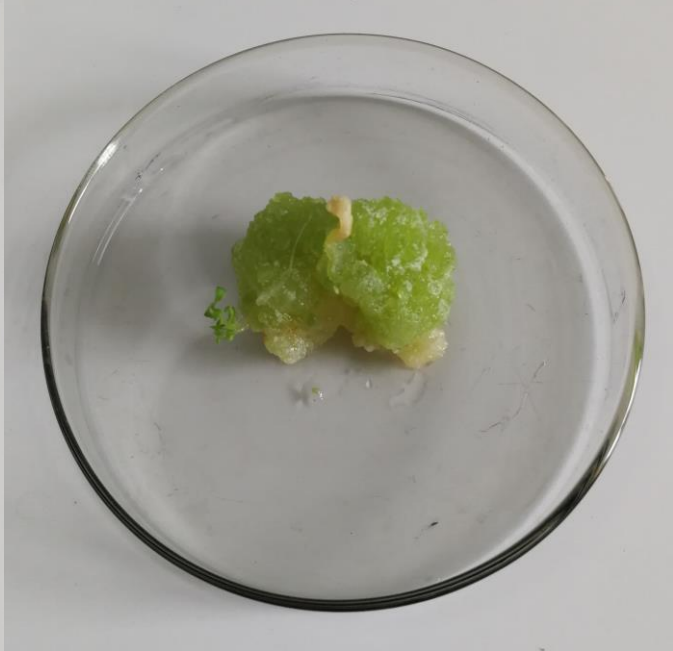

Supplement: Supplementary file 3 — Supplementary Information 3. [file 41598_2021_89294_MOESM3_ESM.pdf]

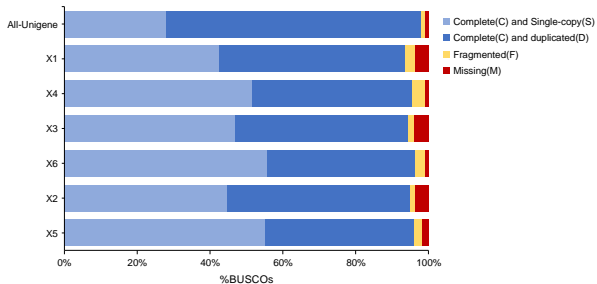

Supplement: Supplementary file 4 — Supplementary Information 4. [file 41598_2021_89294_MOESM4_ESM.pdf]

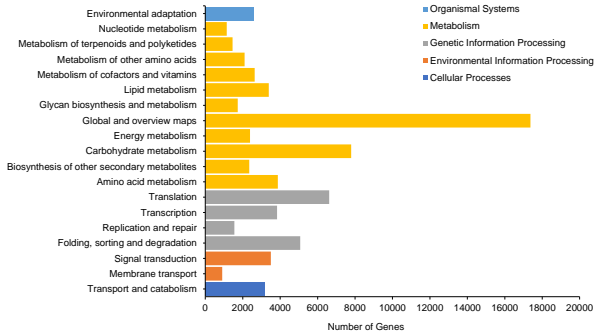

Supplement: Supplementary file 5 — Supplementary Information 5. [file 41598_2021_89294_MOESM5_ESM.pdf]
